# The Chemical Information Ontology: Provenance and Disambiguation for Chemical Data on the Biological Semantic Web

**DOI:** 10.1371/journal.pone.0025513

**Published:** 2011-10-03

**Authors:** Janna Hastings, Leonid Chepelev, Egon Willighagen, Nico Adams, Christoph Steinbeck, Michel Dumontier

**Affiliations:** 1 Chemoinformatics and Metabolism, European Bioinformatics Institute, Hinxton, United Kingdom; 2 Department of Biology, Carleton University, Ottawa, Ontario, Canada; 3 Department of Pharmaceutical Biosciences, Uppsala University, Uppsala, Sweden; 4 Department of Genetics, University of Cambridge, Cambridge, United Kingdom; King's College, London, United Kingdom

## Abstract

Cheminformatics is the application of informatics techniques to solve chemical problems *in silico*. There are many areas in biology where cheminformatics plays an important role in computational research, including metabolism, proteomics, and systems biology. One critical aspect in the application of cheminformatics in these fields is the accurate exchange of data, which is increasingly accomplished through the use of ontologies. Ontologies are formal representations of objects and their properties using a logic-based ontology language. Many such ontologies are currently being developed to represent objects across all the domains of science. Ontologies enable the definition, classification, and support for querying objects in a particular domain, enabling intelligent computer applications to be built which support the work of scientists both within the domain of interest and across interrelated neighbouring domains. Modern chemical research relies on computational techniques to filter and organise data to maximise research productivity. The objects which are manipulated in these algorithms and procedures, as well as the algorithms and procedures themselves, enjoy a kind of virtual life within computers. We will call these *information entities*. Here, we describe our work in developing an ontology of chemical information entities, with a primary focus on data-driven research and the integration of calculated properties (descriptors) of chemical entities within a semantic web context. Our ontology distinguishes algorithmic, or procedural information from declarative, or factual information, and renders of particular importance the annotation of provenance to calculated data. The Chemical Information Ontology is being developed as an open collaborative project. More details, together with a downloadable OWL file, are available at http://code.google.com/p/semanticchemistry/ (license: CC-BY-SA).

## Introduction

Cheminformatics, also known as chemoinformatics, is the field of applied informatics which uses representations of chemical entities, manipulated by software, for the determination and prediction of properties of chemical entities.

There are many areas in biology where cheminformatics plays an important role in computational research. For example, in the elucidation of whole-organism metabolism and metabolic processes: metabolite databases and computational processes for metabolite identification require extensive use of cheminformatics libraries [1]–[4]. Another prominent application of cheminformatics in computational biology is in the understanding of protein-ligand binding patterns, such as are investigated in proteochemometrics [5] and more classical quantitative structure-activity relationship (QSAR) studies [6] which may find protein-protein interaction inhibitors [7]. One critical aspect in the application of cheminformatics in these fields is the accurate exchange of, integration of, and annotation of data [8], [9], for which tasks an ontology such as that presented in this work is crucial.

Cheminformatics has been one of the earliest success stories for the development of novel informatics methods to enhance and supplement the traditional scientific experimental and laboratory-based methods [10], [11]. While the main focus within bioinformatics is on sequence data, in cheminformatics the focus is at the level of atoms and bonds. The chemical graph formalism – in which chemical entities are described in terms of nodes, which correspond to parts such as atoms, and edges, which correspond to bonds – has been widely adopted for denoting the atomic composition and connectivity in chemical entities [10]. Large volumes of data on chemical entities, represented and exchanged in what have become a standard family of formats based on the underlying graph formalism, have been accumulated by commercial databases such as the American Chemical Society's CAS database [12] and the in-house databases of big pharmaceutical companies such as Roche [13] and Novartis [14]. More recently, chemical data has been made freely available – originally motivated by the needs of the bioinformatics research community as it moved towards a whole-systems research perspective – in freely available and public domain databases such as PubChem [15], ChEMBL [16], and ChEBI [17].

The reliable link between chemical structures and chemical properties facilitates research into algorithms and techniques which operate on these structural representations and produce reliable predictions of properties [18]–[20]. This allows, among other applications, computational *screening*, which is the pre-selection of interesting structures for given purposes from the large chemical libraries. Surrounding these innovations and applications, an extensive domain-specific *terminology* has grown which names and describes these chemical information formats, properties, algorithms, and techniques.

However, as is often the case during the development of a new scientific discipline, this terminology has been developed and formalised by many different groups in many different publications and other forms of communication, creating *redundancy*, *ambiguity* and ‘*silos*’ in the eventual terminological system. While this was not so much of a problem as long as all chemical data was locked away behind commercial firewalls, and each individual company working with chemical data had the task of standardising its own internal terminology, in recent years the tide has started to shift towards open data and open algorithms and toolkits in the chemistry domain. In particular, we are seeing the advent of the Semantic Web [21], a set of standards for representing, publishing, sharing, reusing, querying and reasoning about data using web technologies. Cheminformatics data is being brought onto the semantic web in larger and larger volumes [22]. Bringing data onto the semantic web allows it to be used for data-driven research remotely, without the data having to be downloaded and stored locally on the researcher's machine. Semantic web-enabled software fetches desired data from distributed repositories that support cross-resource query answering over heterogeneous data sources.

### Motivation and overview

A key challenge which arises from this novel environment, as compared to traditional in-house data-warehouse approaches, is managing the heterogeneity of publicly available data with special considerations for provenance and reproducibility of data dependent computational experiments [23], [24]. Terminological ‘silo’ problems hinder progress in enabling federated data-driven research on the semantic web in two ways:

- Firstly, different terminologies may refer to the same data with different labels or identifiers. These different labels obscure the fact that the data is comparable and should be integrated, thus ‘hiding’ portions of the data from algorithmic processes of extraction.
- Secondly, multiple implementations of an algorithm may use the same terms, they can produce different outputs due to heuristics, optimizations, errors or outright differences in the interpretation of the terms. This can lead to incorrect deductions when the results of calculations are made available under the same terminological label without further provenance as to which implementation was used to generate the data.

An emergent and rapidly growing solution to terminological ‘silo’ problems on the web is found in the form of logic-based *ontologies*, which are formal representations of objects and their properties within a logic-based ontology language [25]. Many such ontologies are currently being developed to represent objects in all the domains of science. Ontologies formalize the meaning of terms used in a domain, and provide (or at least aim to provide) clear human-readable definitions that disambiguate term usage, along with logical axioms that allow automated reasoning. They enable consistency checking, classification, and query answering over knowledge of a particular domain, enabling intelligent computer applications to be built which support the work of scientists within the domain of interest and across interrelated neighbouring domains. Ontologies are developed and exchanged in a shared ontology language such as the Web Ontology Language (OWL) [26] or the Open Biomedical Ontologies (OBO) format [27].

While ontologies provide formal meaning to terms in a vocabulary, community-based ontologies aim to address the diverse requirements of the members of a community, thus promoting convergence on meaning over a more comprehensive vocabulary than individual efforts might provide. With this in mind, several groups who have independently developed terminologies in the domain of chemical information, have formed a semantic chemistry working group [28]. Through this working group we are developing a unified, coherent ontology to encode the terms, definitions, and logical axioms of chemical information entities, the *Chemical Information Ontology* (CHEMINF) [29].

Cheminformatics data that is brought onto the semantic web is derived from a variety of sources, including direct experimental measurement, algorithmic approximation, and model-based prediction. Approximations and predictions can serve as a guide in the absence of experimental verification of property values. In some cases, such predictions may come close to the experimental values; in other cases they may be far off due to the weakness of the correlation between the best algorithm available to perform the calculation and the actual values. Both measurement and prediction of property values are ways to derive information about chemical or biological properties and represent them in such a fashion that they can be accessible for research which furthers the understanding of biological phenomena. Properly reproduced on the semantic web, such values can be used and reused in multiple scientific analyses and data-driven research projects. Reproducibility of results is of key importance in the scientific method in use across many domains. When such research makes use of data originating from the semantic web, this highlights the importance of maintaining the *provenance* of the information – from detailing the algorithm which was used to generate calculated property values to the specified running parameters and the version of the software implementation.

Our goals in the development of the CHEMINF ontology are thus twofold:

1. Create a reference for the definition and disambiguation of terminology in use in the cheminformatics domain.
2. Provide a framework for the automatic integration of data on the semantic web, including annotation of provenance (for reproducibility), automatic reasoning for classification of data, and query support through semantic web technologies such as SPARQL [30].

In this paper, we present the background, theory, structure and rationale of the CHEMINF ontology. We describe the content of the ontology, both in terms of the class hierarchy and the relationships used to axiomatise the complex interrelationship between algorithms, data types, data formats, procedural parameters, and files stored on computers. Finally, we illustrate an application scenario which makes use of the CHEMINF ontology.

### Background

#### Chemical graph theory, descriptors and QSAR

**Figure 1:**
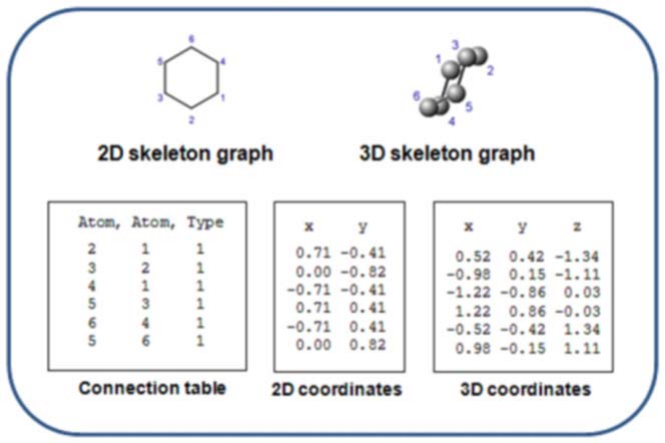
The chemical graph representation of cyclohexane. The chemical graph illustrates the atoms and bonds within a chemical entity, with the exception of hydrogen atoms and their accompanying bonds, which are commonly left implicit since their presence can be deduced from the remainder of the skeleton of the molecule. The graph is illustrated both in 2D and in 3D with the accompanying connection table and coordinates.

Mathematical graph theory, which studies the properties of connected objects, has found many applications in chemistry [10]. Chemical graphs can be used to represent many chemistry-relevant entities including molecules, reactions and clusters. The molecular (or constitutional) graph describes the atomic connectivity within a molecule in terms of nodes for the atoms or groups within the molecule, and edges for the (usually covalent) bonds between the atoms or groups. Although the graph formalism, strictly speaking, represents only the constituents and their bonds, it is usually extended to include other information such as idealised 2D or 3D coordinates for the atoms, and bond order (single, double, triple) and quality (e.g. aromatic). When computational efficiency of molecular graphs must be maximized, non-chiral hydrogen atoms and the edges linking them to their nearest neighbouring atoms, are not explicitly included since the presence and location of hydrogen atoms in the molecule can be inferred from the type, connectivity and charge of the remaining atoms. Such hydrogen-suppressed chemical graphs are called *skeleton graphs*, and these form the most common format for basic chemical information storage and exchange. Figure 1 illustrates the chemical graph for a molecule of *cyclohexane*. Note that the graph includes only carbon atoms as nodes, while in reality cyclohexane molecules have two hydrogen atoms for every carbon atom, with molecular formula 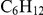. The graph is illustrated both in 2D and in 3D with the accompanying connection table and coordinates.

From these chemical graphs, many molecular properties, such as mass, charge, and shape, can be determined computationally. An example of such a property is the *logP*, which is defined as:

**Definition 1** *The logP is the logarithm of the octanol-water partition coefficient, which is determined from ratio of the molecules dissolved in octanol to those dissolved in pure un-ionized water upon mixture equilibration.*

Such properties are strongly linked to the activity of the molecules within living systems [20], [31]. *In silico* research exploits these associations to make computational predictions of the activity of molecules which have not yet been synthesised, in order to decide which molecules should be synthesised for bench research, and to allow the computational screening of known molecules for new targets to reduce the costs of screening by pre-filtering the molecules which are to be included in the screen. The more effectively such properties can be predicted computationally, the more effective *in silico* research can become.

Quantitative Structure-Activity Relationship (QSAR) descriptors are calculated numeric values, based on structural aspects of a molecule, which can be mathematically correlated with the activity of the molecule [32]. A chemical descriptor can be defined as:

**Definition 2** *A chemical descriptor is the final result of a logical or mathematical procedure which transforms chemical information from a symbolic representation of a molecule into an useful number or the result of some standardized experiment* [*33*] *.*

The field of QSAR descriptors is a very active area of research, with the goal of discovering better performing QSAR descriptors in terms of predicting certain kinds of bioactivity, such as toxicity [34]. Different kinds of QSAR descriptors have been developed which make use of different aspects of the structural information of the molecule, such as atomic descriptors which depend on the atoms in the molecule; connectivity-based or topological descriptors which depend on the connectivity of atoms and bonds within the molecule; and geometrical descriptors which depend on aspects of the three-dimensional shape of the molecule, among others.

#### OBO Foundry, BFO and IAO

The OBO Foundry [35] is an organisation which is coordinating the development of a suite of interoperable reference ontologies for scientific application domains such as biology and medicine, centered around the popular Gene Ontology [36]. As part of this coordination effort, the OBO Foundry requests that prospective member ontologies strive to follow a set of shared, community-agreed guidelines to facilitate orthogonality between the ontologies that are developed and to ensure standard practices of evolution of ontologies are followed. Ontologies which are submitted to the OBO Foundry are first admitted to the OBO Library. They then undergo a peer review process, and if the outcome of this review process is that they display a substantial level of compliance with these guidelines, they are then included as OBO Foundry ontologies. The full list of current OBO Foundry and OBO Library ontologies is available at http://www.obofoundry.org/.

The Basic Formal Ontology (BFO) [37], [38] is an upper level ontology for the biomedical domain. Upper level ontologies contain domain-independent, foundational entities. Other upper-level ontologies include the Descriptive Ontology for Linguistic and Cognitive Engineering (DOLCE) [39] and the General Formal Ontology (GFO) [40]. The alignment of multiple ontologies beneath a shared upper level ontology provides a common framework which supports ontology development through the provision of a foundational structure from which domain-specific entities can be derived [41], [42]. We will focus on the BFO in this paper since that is the upper level ontology adopted by the OBO Foundry, however, alignment of CHEMINF with alternative upper level ontologies is in principle possible and will be the subject of future work. BFO makes foundational distinctions between *continuants* (objects which endure through time, such as humans and trees) and *occurrents* (objects which exist in time, such as events and processes); and between *dependent* and *independent* entities (independent entities can exist by themselves, such as humans, but dependent entities require the existence of another entity for their own existence, such as colours). (A similar top level distinction can be found in DOLCE; GFO additionally distinguishes at the top level between sets and items, and between categories and individuals.) Together with the Relation Ontology (RO) which provides fundamental relations between BFO entities [43], BFO provides a common organising high-level framework for the development of domain ontologies.

Information entities, such as those which we include in the chemical information ontology, are a kind of dependent entity. Dependent entities are those which cannot exist without a bearer. For example, colour is a dependent entity since there can be no colour without there being something that it is the colour of. Similarly, information entities are dependent entities – there can be no information without it being stored somewhere, such as on my computer – but this dependence functions in a slightly different fashion to that of hair colour, since information can be *copied*. BFO distinguishes *specifically* dependent entities, which cannot be copied, from *generically* dependent entities, which can. Information entities are thus kinds of *generically dependent continuant* in BFO.

The domain of information entities *in general* is being addressed by the *Information Artifact Ontology* (IAO) [44], another project under development within the OBO Foundry community. While the IAO is concerned with the domain of information entities in general across all domains, and without delving into the specific terminologies of any one scientific domain, our work is focused on only those information entities of relevance in the domain of cheminformatics. As such, the CHEMINF ontology falls hierarchically beneath the IAO, as we will illustrate in the next section on the structure of the ontology.

Crucial to the IAO definition of information entities is that they are *about* something, which is encoded in the IAO as:

*Information content entity* 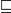 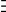 **is_about**.*Entity*

For example, a name is an information entity, and a name *is_about* the thing that it is a name of.

This ‘aboutness’ relationship is problematic in some cases, for example, in chemistry, information content entities may be created for chemical entities which do not yet exist during the course of *in silico* research [45]. We recognise that this problem is a contentious one in the bio-ontology community at the moment (see, for example, the discussion on the OBO Foundry email mailing list, entitled ‘Ontological Realism and OBO Foundry Criteria’, dated July 14, 2010), but as this relation is defined in the IAO with regard to *all* information entities, rather than just *chemical* information entities, we do not attempt to take up this debate further here, but the interested reader can see [46].

#### Classes and individuals

Ontologies consist of, on the one hand, general entities called *classes* and the relationships between them (properties), and on the other hand specific entities called *individuals*, that belong to classes. For example, a particular person called Mary is an individual, and she belongs to many classes including the class of all humans. Classes are predominantly the subject matter of ontologies, as the ontologies are being developed to support multiple annotations of scientific data [35], and the same individual, say Mary, is not often the subject of multiple scientific experiments, but rather it is classes such as *Human* which are the subject of multiple scientific experiments, and the actual individuals who participate in the experiments are exchangeable as long as they are of the right type. (Although, exceptions do exist. For example, *standard units* may be best represented as individuals in ontologies.)

However, with respect to information entities, it is not as straightforward to decide what to model as classes and what as relevant individuals. This is because information may be copied and transferred between bearers, in a way that material individuals cannot. A single computer file can be copied between multiple computers. Should we model it as an individual? Or should we rather model a class of files containing the same information content? The Blue Obelisk Descriptor Ontology BODO [47], for example, models chemical graphs as individuals. However, this prohibits the expression of hierarchical relationships between graphs, even if one of the graphs expresses a more general information content than the other [48]. For this reason, we adopt an approach which does allow for the expression of hierarchical relationships between chemical graphs, and model chemical graphs and other information entities as classes in the CHEMINF ontology.

In the next section we describe the structure of the ontology.

## Results

The chemical information ontology (CHEMINF) is implemented with the Web Ontology Language (OWL2) [49]. Classes in the ontology have identifiers of the form http://semanticscience.org/resource/CHEMINF_XXXXXX, and include labels (rdfs∶label) and definitions (dc∶description). The ontology is versioned using owl∶VersionInfo. All CHEMINF resources are Linked Data nodes, and their URIs are dereferencable.

The expressivity of the ontology is 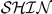
(**D**), thus contains atomic concepts and roles, transitive roles, conjunction, disjunction, existential and value restriction, role hierarchies, inverse roles, number restrictions and datatypes [50]. CHEMINF extends the Ontology for Biomedical Investigations (OBI) [51], the Information Artifact Ontology (IAO) [44], the Relationship Ontology (RO) [43] and the Basic Formal Ontology (BFO).

### Scope

The ChEMINF ontology includes entities such as:

- Chemical graphs, and various formats for encoding them.
- Chemical descriptors, with definitions and axioms describing what they are specifically about.
- Specifications for certain descriptors.
- Algorithms and their software implementations and axioms describing their inputs and outputs.
- Chemical data representation formalisms and formats.

Additionally, we have identified a hierarchy of chemical qualities, which are needed to specify exactly which quality a chemical descriptor is describing. However, in keeping with the OBO Foundry's principle of orthogonality of ontology application domains, we have submitted these chemical quality terms to the Phenotype Quality ontology (PATO) [52].

We explicitly exclude from the scope of CHEMINF:

- Actual chemical entities, parts, ions, groups, etc which are included in the ChEBI ontology [17].
- Any aspects of protein or nucleotide sequence information which are included in the Sequence Ontology [53].
- We include named algorithms, but do not give the algorithmic steps. The relevant paper describing the algorithm is linked to from the definition where possible.
- Similarly, for format specifications (such as Chemical Markup Language (CML) [54]), we provide a citation rather than reproducing the detail of the specification.

The ontology is licensed as Creative Commons Share-Alike By Attribution and is freely available from the Google Code project site http://semanticchemistry.googlecode.com. To preserve modularity and ease of maintenance, the ontology consists of multiple files, with one such separate file, for example, providing mappings to the Blue Obelisk Descriptor Ontology. These separate files are referenced from the primary ontology file cheminf.owl using the OWL import mechanism.

### Ontology content and organisation

**Figure 2:**
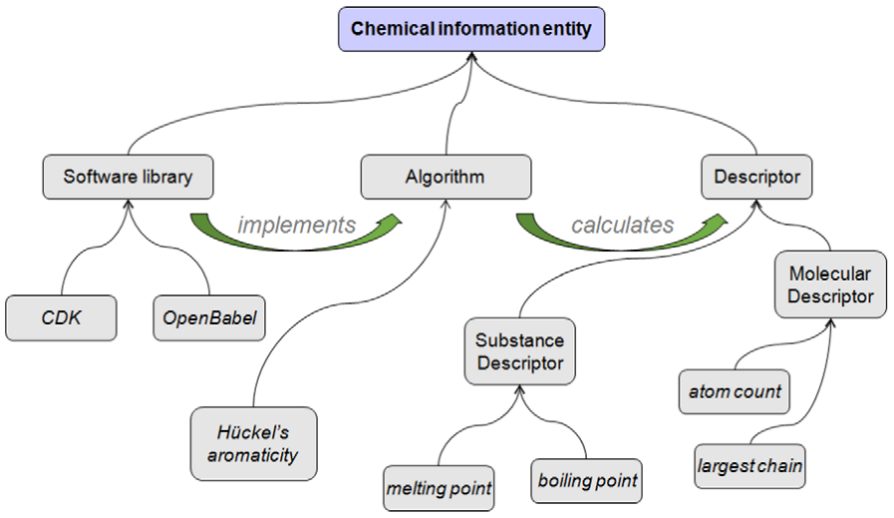
An overview of the content of the CHEMINF ontology. The diagram gives a schematic illustration of the ontology content, which can be divided into named descriptors, named algorithms which calculate descriptors, and software libraries which contain software modules that implement algorithms.

Figure 2 provides a schematic overview of the content of the CHEMINF ontology. The basic content of the domain terminology can be divided into named descriptors, named algorithms which calculate descriptors, and software libraries which contain software modules that implement algorithms.

Not illustrated in this diagram are the processual executions of the software implementations, as these fall within the hierarchy of processes rather than information entities. The link between processes which are software executions, and the software implementation that is executed, is that the process has the software as *agent*.

The key entities in our ontology, situated beneath their appropriate superclasses in the referenced ontologies, and including their number of subclasses, are given in Table 1. The most well-developed branch of the ontology is the chemical descriptor branch, as we have already included 180 different descriptors in the ontology, including a broad range from simple descriptors such as *atom count* to more complex descriptors such as *topological polar surface area*. Descriptors give information about qualities of chemical entities, and to formalise this association, we have added 50 chemical entity qualities to the quality branch of the ontology, including *polarizability* and *relative permittivity*. Format specifications, such as *MDL molfile* [55] and the Chemical Markup Language *CML* [54], are the next largest branch of the ontology, and finally the algorithm and software implementation branches of the ontology are not yet well developed, although there is an ongoing effort to include all algorithms and implementation details for the Chemistry Development Kit (CDK) [56] in the ontology, and this will be forthcoming in a future release.

**Table 1:** Key entities in CHEMINF ontology and their immediate superclasses.

entity name (ID)
directive information entity (IAO_0000033)
– data format specification (IAO_0000098)
– – *molecular entity information format specification* (CHEMINF_000014) – 21 descendent classes
plan specification (IAO_0000104)
– algorithm (IAO_0000064)
– – *algorithm to calculate a chemical descriptor* (CHEMINF_000144)
software (IAO_0000010)
– *software module* (CHEMINF_000340)
– – *software module to calculate a chemical descriptor* (CHEMINF_000103)
data item (IAO_0000027)
– *chemical descriptor* (CHEMINF_000123) – 180 descendent classes, including:
– – *chemical graph* (CHEMINF_000400)
quality (in BFO)
– *molecular entity quality* (CHEMINF_000031) – 42 descendent classes
– – *chemical substance quality* (CHEMINF_000101) – 5 descendent classes
planned process (OBI_0000011)
– *software execution* (CHEMINF_000138)
– – *parameterized software execution* (CHEMINF_000147)

The key CHEMINF entities are chemical domain specialisations of more general terms in IAO and OBI. They are: chemically relevant format specification, algorithm, software module, chemical graph, and software execution.

The key relations in our ontology are:

- An information content entity *is about* some entity; an entity *is described by* some information content entity. (We note that the inverse of the *is about* relation is considered problematic from an ontological perspective since information is not a property of the thing it is information about. However, we introduce this inverse relation *is described by* here as a convenient shorthand for referring back from an entity to information, where the aboutness is already captured in the ontology.) The IAO *is about* relationship is further specialized into different subrelations, of which one example is the *is quality measurement of* relationship, which relates a measured datum to the particular quality that it is a measurement of. While this relation is close to what we need in order to relate chemical information entities to properties of the chemicals that they are about, we allow chemical information that is both *measured* and *calculated*, and we therefore introduce a distinct relation *is descriptor of*.
- A chemical descriptor *is descriptor of* some specifically dependent continuant (quality or other property); a specifically dependent continuant *has descriptor* some chemical descriptor.
- An information content entity *conforms to* some directive information entity (i.e. specification); the directive content entity *specifies* an information entity.
- An entity *has attribute* some data item; the data item *is attribute of* some entity.

Other relations which we make use of in the ontology including the *bearer of* relation which links independent entities to the dependent entities (such as qualities) which inhere in them, and the *has part* and *part of* mereological relations, which are inherited from the Relation Ontology, and the *has value* data relation which links a data item to its value.

We now discuss the ontology model in more detail for the specific topic areas of format specifications, chemical descriptors, and algorithms and implementations.

### Modelling specifications

In cheminformatics, many information objects are created in order to standardise or *specify* formats for data exchange or the operational requirements of a particular procedure. These information objects have a kind of normative content, creating – in their information content – a requirement on the information objects that conform to them. We model this type of information object as *directive information entity*.

**Definition 3** *A directive information entity is an information content entity that explicitly states essential attributes/requirements for a product or procedure, and may also be used to determine that the product/procedure meets its requirements/attributes.*

One special kind of directive information entity is that which specifies the format for the encoding of information such that it can be encoded and decoded in a standard way. This is a *data format specification*.

**Definition 4** *A data format specification provide directives regarding the syntax of information such that it can be encoded and decoded in a standard fashion.*

**Figure 3:**
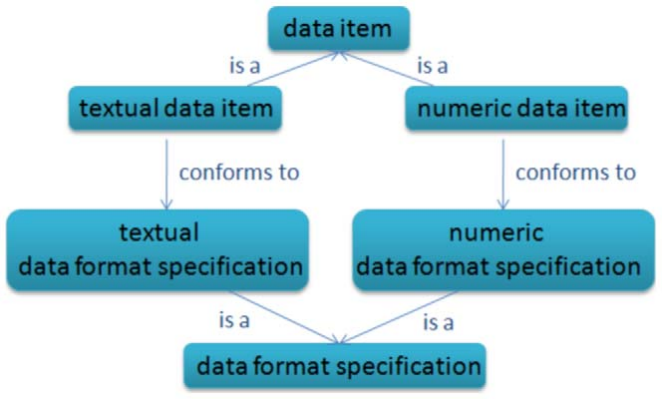
Textual and numeric data format specifications. An example of format specifications are those which constrain the format of a data item to be textual or numeric. In the case of a numeric format specification, only numeric digits are allowed in the data item. Format specifications are essential when designing robust software for complex research pipelines.

Some examples of data format specifications are the MOL and SD file format specifications commonly used for chemical graph storage and exchange [55], the Simplified Molecular Input Line Entry Specification (SMILES) format specification [57], and the basic data format specifications such as integer or numeric which are associated with the input parameters of algorithms, as illustrated in Figure 3.

These data formats are then used in the definition of different types of chemical descriptors.

### Chemical descriptors

The most general type of chemical information entity is that which captures some sort of data about some chemical entity. We use the term *chemical descriptor*.

**Definition 5** *A chemical descriptor is a data item (a quantity or value) whose syntax and semantics conforms to some data format specification and provides information about chemical entities including, but not limited to reactions, substances, molecular entities, and their parts (rings, atoms, bonds, etc).*

Note that the term ‘descriptor’ has a narrower meaning in some cheminformatics communities, i.e. restricted in use to only those descriptors which have numeric values and which can be used in quantitative structure-activity-relationship models. For these types of descriptor, we propose the subtypes ‘numerical chemical descriptor’ which is defined in terms of the data type of the descriptor, and ‘QSAR chemical descriptor’ which is described in terms of the applicable usage of the descriptor.

Chemical descriptors may enumerate material or processual parts, quantify qualities or realizables including dispositional probabilities. For example, a SMILES descriptor, which conforms to the SMILES specification for unambiguously describing molecular structure using short ASCII strings, can be created for aspirin (acetylsalicylic acid, CHEBI:15365) with value CC( = O)Oc1ccccc1C(O) = O.

The following example shows, in Manchester OWL syntax [58], some descriptors (SMILES, InChI and InChIKey) associated with aspirin (acetylsalicylic acid) using CHEMINF:

Class: ‘acetylsalicylic acid’

   SubClassOf:

   has_attribute ‘acetylsalicylic acid InChI’,

   has_attribute ‘acetylsalicylic acid InChIKey’,

   has_attribute ‘acetylsalicylic acid SMILES’

Individual: ‘acetylsalicylic acid InChI’

 Types:

  ‘InChI Descriptor’,

Facts:

  ‘has value’

   “InChI = 1/C9H8O4/c1-6(10)13-8-5-3-2-4-7(8)9(11)12/h2-5H,1H3,(H,11,12)/f/h11H”

Individual: ‘acetylsalicylic acid InChIKey’

 Types:

  ‘InChIKey Descriptor’,

Facts:

  ‘has value’ “InChIKey = BSYNRYMUTXBXSQ-WXRBYKJCCW”

Individual: ‘acetylsalicylic acid SMILES’

 Types:

  ‘SMILES Descriptor’,

Facts:

  ‘has value’ “CC( = O)Oc1ccccc1C(O) = O”

Descriptors for chemical entities often describe aspects of the structure of chemical entities. Structural descriptors have the additional property that, while remaining within the rules of the structural representation formalism, cannot change value without representing a different entity. To put this differently: chemists cannot have a meeting and *decide* to give a different structural descriptor to a particular chemical entity, as they can for a name. The structural descriptor is *constrained* by the format specification and the structure being described. Chemists could, of course, decide to change the format specification, and many new structural descriptors are born through the invention and specification of new formats. Note that in many cases, the structure of a chemical entity may not be known at the time that the chemical is named. In other cases, a structure is presented but is later found to be incorrect, and needs to be revised throughout public databases. Chemical entities are therefore not identical with their structural representations (such as chemical graphs). Indeed, structural representations give a *static* view of the nature of chemical structures, which is an approximation to the actual dynamic reality.

Our model allows the explicit linking not only of a descriptor to the kind of entity it is about (such as a molecule), but also to the particular property of that entity that the descriptor is representing. For example, a charge descriptor is descriptor of the electrical charge quality of a molecule. In this way, descriptors can be grouped together based on the nature of the properties that they describe. However, there are some descriptors for which the exact molecular property that the descriptor is describing is unclear; in these cases we remain agnostic and make no assertion above the claim that the descriptor is about the molecule, with the possibility to pick out those specific attributes which formed the input to the descriptor calculation.

**Figure 4:**
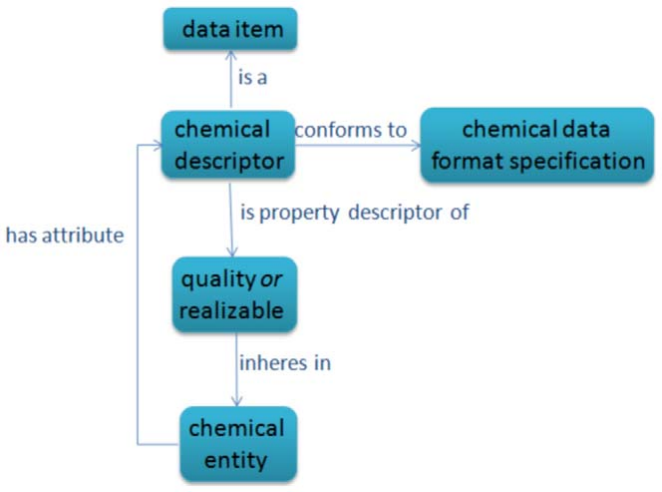
Chemical descriptors. A chemical descriptor conforms to a data format specification. It is about a chemical entity (an example of which might be ‘caffeine’), and is a descriptor of a property of that chemical entity (such as its charge). The descriptor value is linked to the chemical entity in the ontology with the *has attribute* relation.

Figure 4 shows an illustration of the CHEMINF ontology model for chemical descriptors. Chemical descriptors are data items which are about chemical entities. They conform to a chemical data format specification, and they are descriptors of a property (quality or realizable) which inheres in a chemical entity.

Chemical descriptors can be obtained from physical experiments, in which something is quantitatively measured. We say that an experiment involves some chemical substance as input and produces some chemical data as output. For instance, the structure of a chemical substance can be investigated using nuclear magnetic resonance (NMR); this requires as input some chemical substance in buffered solvent within some concentration range and produces as output resonance frequencies.

On the other hand, descriptor values can be generated *in silico* from the analysis of computational representations of chemical entities by software applications.

### Algorithms and software implementations

When using software to predict chemical attributes, software applications consume some kind of data and produce some kind of data. Software, modules and methods are expressed as source code using programming languages that are subsequently compiled into a machine interpretable format. These software methods are specified by one or more algorithms, or sequences of steps. Like format specifications, algorithms are directive information entities.

**Definition 6** *An algorithm is a directive information entity that consist of a finite sequence of instructions to accomplish a task, which may be expressed in pseudocode, textual description, or a process flow diagram.*

Chemical descriptors are distinguished from the algorithms which generate them, although in many cases they share a common name, since algorithms specify procedural information, while descriptors are declarative information. In some cases, the same descriptor can be calculated by several different algorithms.

Named algorithms may have different versions. For example, the Kabsch algorithm for calculating the optimal rotation matrix for alignment of two chemical structures was first presented in [59], and a later correction was presented in [60]. In this case it can be said that there are two versions of the Kabsch algorithm, and it is useful to distinguish these in implementations. To model this scenario in CHEMINF, we create a superclass for the named algorithm and create subclasses for each of the versions. In cases where it is known which version is implemented in a particular library, this can be annotated to the versioned subclass, and in cases where it is not known, the annotation to the parent class can be used instead.

Algorithms are also distinguished from the software which implements the algorithms. This is because it is possible for an implementation to contain errors, or to be more or less faithful to the designed algorithm which it implements. Programming languages have different constructs and performance profiles which lead to subtle differences in different implementations of the same algorithm. For this reason, to correctly associate provenance with calculated descriptor values, we suggest at minimum the annotation of calculated values to the software implementation rather than directly to the algorithm, and preferably with detail about the fully specified process execution, as discussed below in the context of data transformation operations.

Software implementations can be stand alone single software methods, or they can be packaged into software libraries. For example, the Chemistry Development Kit (CDK) [56] is a software library containing a wide collection of modules for manipulating chemical information. Software implementations are associated with a programming language, we say that the implementation *has agent* the programming language.

**Definition 7** *A software implementation is a machine-executable set of instructions in some programming language. Software implementations generally belong to some named library, which is a collection of related software modules. Individually executable methods or components of a software implementation take input parameters, execute some operations using such input values, and produce some output parameters.*

Considering the software maintenance lifecycle, most software implementations are continuously evolving. Different versions of software arise from this maintenance cycle, each being a different manifestation of the relevant source code, in that they are variants of each other.

**Figure 5:**
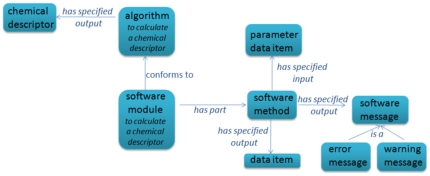
Algorithms and software implementations. Algorithms are differentiated in the ontology from the software which implements them. The same algorithm may, for example, be implemented in several different programming languages. The smallest unit of software which we identify is the software method. Methods have parameter data items as input and generate resulting data items as output. Software methods also may generate warning or error messages. Multiple software methods are grouped together into a software module. A software module may conform to an algorithm which has specified output a particular descriptor.

Figure 5 shows the CHEMINF object model for algorithms and software implementations. Algorithms have specified output a particular chemical descriptor. A software module, which consists of one or more software methods, conforms to an algorithm. Each software method has zero or more input parameters and zero or more output data items (which may themselves become parameters as input to another software method). In addition to having data items as output, a software method may also raise software messages as output – for example, error or warning messages.

When software is actually executed within some pipeline or towards some objective, its execution is a process. The outcome of this process depends on many factors in the execution environment, including the hardware platform on which the process is executed and the operating system and other supporting libraries which are installed on that platform. For example, a *data transformation* operation is the execution of a software module with specific parameters as inputs.

**Definition 8** *A data transformation is a planned process that realizes some agent-specified objective. It requires the software which is being executed and the hardware on which it is executed as participants, and may require data items as input, and may produce data items as output.*

**Figure 6:**
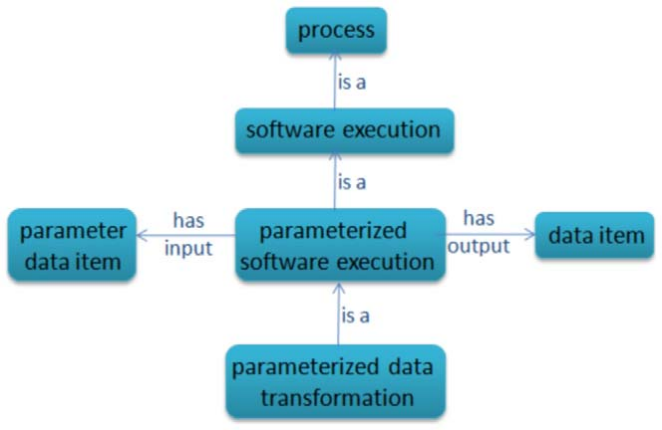
Data transformation. A data transformation is an example of a parameterized software execution. A software execution is differentiated from a software method or module in that the software execution is a single process which has concrete parameter values as input. On the other hand, a software method or module may be executed over and over with many different parameters.

Figure 6 shows the CHEMINF ontology model for data transformation operations. Since the behaviour of a data transformation operation is often dependent on the value of the input parameters, for full metadata about calculated values it is important to associate them with the fully specified process execution.

### Classification of entities within the ontology

In the CHEMINF ontology, we create different axes of classification, such as the axis of classification based on the type of entity that a descriptor is about, through the use of *defined classes*, i.e. classes which are fully logically defined through the specification of necessary and sufficient conditions. These logical definitions allow the use of a reasoner to compute subsumption (classification) beneath differently defined parent classes. This avoids the need to maintain separate classification hierarchies by hand in order for the result to include classification along multiple possibly orthogonal axes.

Examples of classes which we have defined using necessary and sufficient conditions in this fashion are *chemical substance descriptor* and *molecular entity descriptor*, which are defined as those descriptors which are *about* chemical substances or molecular entities respectively. Note that a chemical substance is a bulk collection of molecular entities, such as a portion of water compared to an individual water molecule.

**Figure 7:**
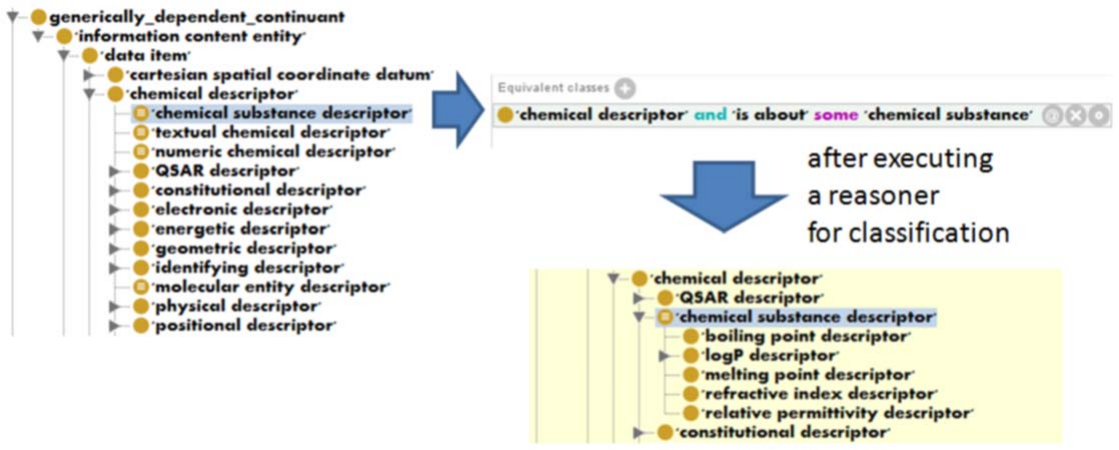
Automatic classification based on logical definitions. The diagram illustrates the use of logical definitions in terms of necessary and sufficient conditions (equivalent classes), which are then used by a reasoner (in this case Fact++) to derive the subsumption hierarchy for member classes based on their properties.

Figure 7 shows an extract from our ontology before and after the reasoner has performed a classification task, illustrating the calculated subsumption relationships. The class ‘chemical substance descriptor’ has no asserted children, but after reasoning, the children are inferred based on the information encoded for each descriptor in the ontology.

## Discussion

We have presented an ontology for the domain of chemical information entities, with primary application to the disambiguation of data types in data integration tasks and the assocation of provenance with data especially in the context of the semantic web.

### Related work

Early ground work in the area of the ontology of chemical information was laid by Gordon in his 1988 series entitled *Chemical Inference* [61]. Here, a logical treatment of chemical entities, properties and relations (such as tautomerism) is laid out, and our work derives much from that treatment. We also rely on standard elements of chemical graph theory as presented in [10].

The CHEMINF ontology describes chemical descriptors, software and algorithms. As such, we investigated in which other formalisms such objects are described. Chemical entities are often described and exchanged in the MDL SDF file format [55], which allows content providers to append descriptor values as free text under free text headers, and the CML file format [54], which is as per SDF but further enables such values to be explicitly annotated using XML dictionaries. While these formats allow for exchange of descriptor values according to a set dictionary, they offer no information as to the descriptor definitions, generating software, and ontological classification.

Closely related to our work on the ontology of chemical information is work on the chemical semantic web, in which data is being brought online on the Internet in the form of Resource Description Framework (RDF) ‘triples’. The RDF vocabulary Comb*e*Chem [23], [24] was used to capture some aspects of chemical structure and identity with an emphasis on provenance as well as state-dependent (those that depend on pH, temperature, pressure) and state-independent properties (identifiers, molecular weight). CHEMINF does not explicitly distinguish between state-independent and state-dependent properties in this fashion, rather, we have several different categories of descriptor which have different dependence conditions, including ‘identifying descriptor’, ‘physical descriptor’ and ‘electronic descriptor’. Furthermore, the Comb*e*Chem RDF schema (i.e. their ontology) only defines a vocabulary with basic types such as *Molecule* and *Property*, and the different types of property are associated with different predicates such as has-name and has-SMILES for name and SMILES respectively. Going this route would require adding a predicate (in OWL, an object property) for every different descriptor, and is thus much more difficult to maintain and develop related applications around than the approach of allowing an extensible hierarchy of descriptors such as we adopt in CHEMINF.

To demonstrate the potential of semantic web technologies for semantic data integration, Konyk *et al.* focused on representing chemical structure and being able to associate simple computed attributes [62]. This work featured queries that involved automated reasoning of OWL ontologies for chemical functional groups across RDFized versions of PubChem, DrugBank [63] and Wikipedia. More recent work in providing chemical structure and properties in RDF has been conducted by Willighagen *et al.* [64], in which an RDF schema is provided not only for molecules and properties, but also for descriptor values and implementations, with a model provided that links implementations to vendors and parameters. The descriptor types which are supported by this implementation are those described in the Blue Obelisk Descriptor Ontology (BODO) [47], [65]. BODO is available in OWL format, and indeed some terms are shared between the two projects (and mapped accordingly), however, BODO does not provide a formal axiomatization of descriptor types, relying rather on a hierarchy listing descriptor names, and listing the specific descriptors provided by specific vendors as instances of these general types.

Our work is thus an extension of these earlier RDF offerings in providing an ontology for the classification and axiomatisation of such properties, while keeping to the more valuable elements of the model such as allowing explicitly for different vendors providing different implementations and explicitly stating the parameter values used in calculations.

### Applications and evaluation

The semantic web makes it possible to publish, share and integrate data online. However, making data available on the semantic web is only the first step towards the vision of seamlessly distributed and integrated data being available for application consumption. In a data warehousing approach without the semantic web, each application downloads and consumes the source data it requires in whichever proprietary format that data is made available in. Extensive in-house processing is required to transform all the disparate sources of data into a common format which allows for comparison and integration, and custom rules are required for performing such integration. This leads to a huge maintenance overhead, since in order to keep the data up-to-date, the process must be regularly repeated, and application changes are required every time something changes in the representation format used in any of the source databases.

The semantic web approach is to replace custom in-house data warehousing with distributed data sources which are integrated on the fly by applications as they perform their required functions. The data is thus consumed when it is needed, and local copies are not maintained, avoiding the large maintenance and redundant storage overheads associated with the data warehousing approach. One of the requirements for such an endeavour to work is that all the data is made available in a standard format that can be processed in a uniform fashion - which is RDF in the context of the semantic web. Data provision in RDF addresses the *syntactic* aspects of the challenge of on-the-fly data integration. However, this goes only a part of the way to resolve the underlying issue, since there is also a *semantic* challenge in data integration, since data items may be named and identified differently by different data providers (or even by the same data provider in different contexts, although we hope that internal standardisation efforts will go some way to alleviate this). For example, one database may provide IUPAC names for chemical entities in a field entitled ‘iupacName’, while another may provide the IUPAC name in a field entitled ‘systematic name’. Without human intervention, it is impossible for computers to integrate these two data items - i.e. to unify them in the resulting application view which has consumed the data from both sources.

At the very least, community standards for the different identifiers that are to be used for different types of data are required so that the data from different data providers can be automatically unified where applicable. A ‘flat’ list of standardised identifiers for different types, such as a dictionary or controlled vocabulary can provide, would be sufficient for this purpose. Since data that is brought onto the semantic web is linked by relationships, e.g. the name is associated with the chemical that it is the name of, a similar need for standardisation applies to the relationships used and to the types of entity that can be related by those relationships. This type of specification goes beyond that which can be provided by a flat dictionary, thus a schema definition language such as RDFS is required. However, logic-based ontologies such as CHEMINF which are represented in OWL provide both of these functions and have an additional benefit besides: they allow for complex *reasoning* which serves the diverse purposes of consistency checking (i.e. automated checks for modelling and data errors), classification (automated computation of complex hierarchies based on the specified logical definitions), and sophisticated question answering.

The use of OWL ontologies for the annotation of data brought onto the semantic web thus supports and facilitates the semantic web vision in several different ways - through standardisation of identifiers, through standardisation of modelling schemas, and through the ability to perform logic-based reasoning for classification, consistency checking and question answering. We have already described the classification of entities in the ontology through use of the logical definitions, and consistency checking is regularly used as part of the ontology development process to ensure that released versions of the ontology are error-free. Here, we further describe our efforts to annotate publicly available semantic web data with CHEMINF, and detail an example of question answering on annotated data using reasoning over the ontology.

#### i) Annotation of publicly available semantic web data

CHEMINF has been used to standardize the annotation of SMILES and InChI strings in the recently introduced RDF version [66], [67] of the ChEMBL database [16], labeled ChEMBL-RDF. This allows software such as Bioclipse [68] to automatically discover molecular structure information available via SPARQL end points. For example, the following SPARQL query retrieves the number of molecules in a remote end-point.

PREFIX cheminf: <http://semanticscience.org/resource/>

SELECT count(?molecule) as ?moleculeCount WHERE {

 ?molecule cheminf:CHEMINF_000200 [

  a cheminf:CHEMINF_000113;

   cheminf:SIO_000300 ?inchi

   ]

   }

Previously, we have demonstrated the use of Bioclipse and SPARQL to extract QSAR datasets from the ChEMBL database [66]. We have now further standardized this workflow by using the fact that ChEMBL-RDF now expresses molecular SMILES representations using CHEMINF. This way, we can query, for example, all molecules for a particular assay with 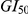 values (concentration that results in 50 We have additionally used CHEMINF to annotate descriptors in an RDF version of the ChEBI database [17]. The RDF is available at http://s3.semanticscience.org/ and a faceted browsing interface is available at http://bio2rdf.semanticscience.org:8035/fct/. The SPARQL endpoint is at http://bio2rdf.semanticscience.org:8035/sparql/. All of the descriptors which are included in ChEBI have been annotated with their respective CHEMINF values, thus unambiguously identifying their type.

Integration of data from disparate data sources and several domains, as well as subsequent querying, as enabled by CHEMINF, has recently been demonstrated with the Chemical Entity Semantic Specification formalism (CHESS) [69]. In the coming decades, we expect Semantic Web technologies to play an increasingly important role in the representation of chemical information, leading to the appearance of a large array of representational formalisms similar to CHESS. However, so long as these formalisms adhere to the common CHEMINF ontology just as CHESS did, we expect that this new chemical data representation formalism divergence shall not translate into fragmentation of chemical databases and difficulties in the automated federation of chemical information. On the contrary, adoption of CHEMINF will allow disparate disciplines of chemistry (and beyond) to interlink and become amenable to interdisciplinary querying.

#### ii) Question answering

In order to illustrate question answering over an annotated knowledge base, we transformed a set of 90 antidepressant drug molecules from PubChem [15] into a knowledge base together with calculated descriptors from three different software libraries, as described in the Methods section of this paper. Provenance was captured in terms of the software (and version) plus parameters used to generate the descriptors. The full knowledge base is available for download from: http://www.ebi.ac.uk/hastings/downloads/cheminfpopulated.zip.

We queried our sample knowledge base using the DL Query tab of Protégé. A query to evaluate drug-likeness according to the Lipinski Rule of Five (described in [70]) is shown below (Manchester syntax again).

‘chemical entity’ and ‘has attribute’ some

 (‘molecular mass’ and ‘has value’ some double[< = 500.0 ])

and ‘has attribute’ some

 (‘XLogP descriptor’ and ‘has value’ some double[<5.0])

and ‘has attribute’ some

 (‘hydrogen bond acceptor count’ and ‘has value’ some int[< = 10])

and ‘has attribute’ some

 (‘hydrogen bond donor count’ and ‘has value’ some int[< = 5])

This query retrieves from the knowledge base all those chemical entities that are compliant with the Rule of Five, that is, they have molecular mass no more than 500; they have an XLogP of less than 5; they have a hydrogen bond acceptor count of not more than 10 and a hydrogen bond donor count of not more than five.

**Figure 8:**
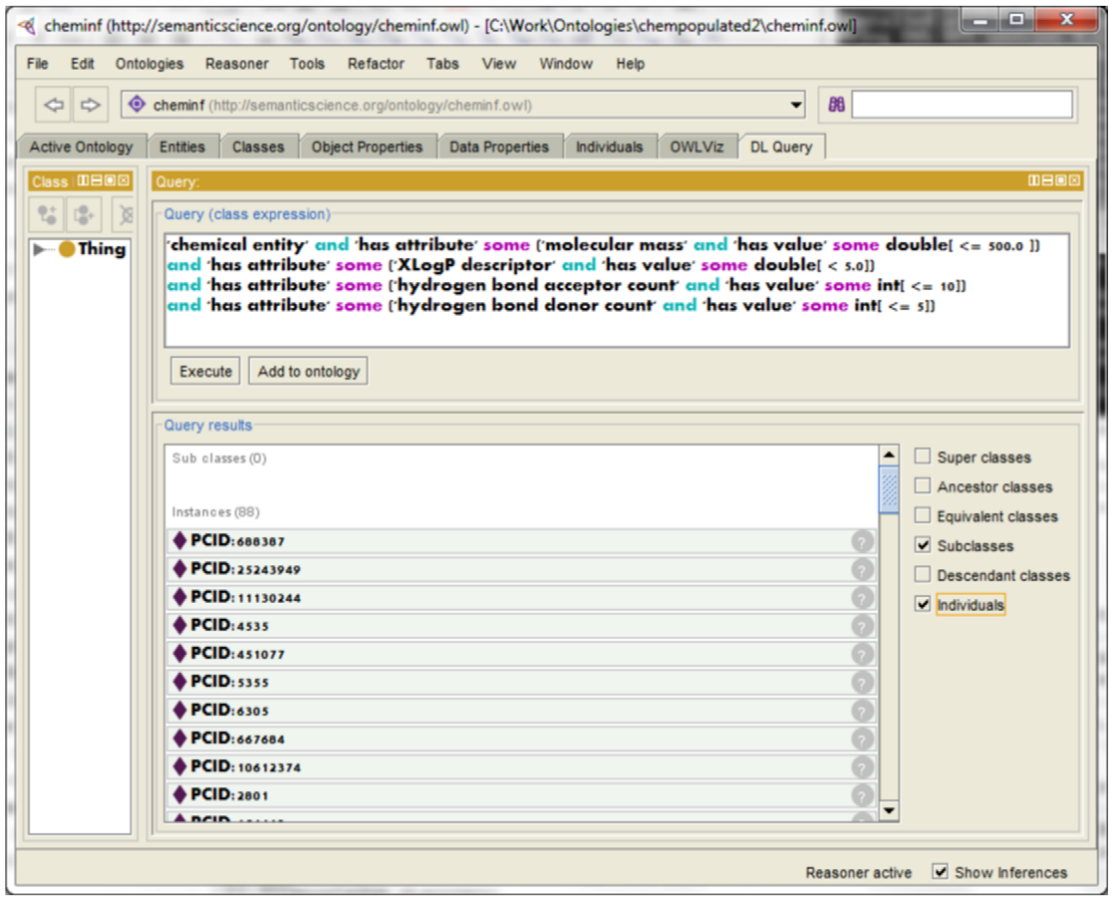
Lipinski query results. The diagram illustrates the Protégé query tab and the results of executing a Lipinski drug-likeness query on the generated CHEMINF knowledge base.

In our knowledge base of 90 antidepressants, we would expect that many of them, being known drugs, would be compliant with the Rule of Five, and indeed this is the case: 88 molecules match the query. The result of applying the query in the DL query tab are shown in Figure 8.

Another query example is to retrieve all the descriptors calculated by a particular software library. We can use the following query to retrieve this:

‘data item’ and ‘is output of’ some

(‘software execution’ and ‘has agent’ some ‘Chemistry Development Kit’ )

In our annotated knowledge base, this query returns 361 instances, which include the CDK-generated XLogP and HBond descriptors.

The above queries have been executed using the DL query tab in Protégé, which allows the construction of queries which are evaluated using *instance checking* (for individuals) and *classification* for subsumed classes. This type of querying is very useful against knowledge encoded in OWL ontologies, but has limits when compared to the expressivity of querying in the Semantic Web context, in which SPARQL is usually used as the query interface. However, SPARQL is not OWL-semantics aware, so it is not possible to query OWL constructs using SPARQL. A query mechanism based on SPARQL but which does interpret OWL-DL semantics is SPARQL-DL [30], provided as a query interface within the Pellet reasoner [71].

SPARQL-DL is useful for, like SPARQL, allowing the tabulation of results based on querying the knowledge base – that is, not only finding which entities match a given class description, but retrieving ordered lists of attributes of entities, like a database query. For example, the following SPARQL-DL query retrieves the names (within the knowledge base) and associated descriptors and values which were calculated using OpenEye. The key difference to a straightforward SPARQL query in the below is that SPARQL-DL is able to interpret the semantics of the rdfs:subClassOf operator and check this against the OWL hierarchy.

select ?compname ?desctype ?descname ?descvalue

WHERE {

 ?compound ci:CHEMINF_000200 ?X ;

   rdfs:label ?compname.

?X rdf:type ?Y ;

  ci:CHEMINF_000012 ?descvalue ;

  rdfs:label ?descname ;

  ci:CHEMINF_000606 ?Z.

?Y rdfs:subClassOf ci:CHEMINF_000186 ;

  rdfs:label ?desctype.

?Z ro:has_agent ci:CHEMINF_000267.

}

In this query, the prefix ‘ci’ refers to an entity in the CHEMINF ontology and ‘ro’ the relationship ontology. The query retrieves a compound (*?compound*) which has a label (*?compname*) and has an attribute (CHEMINF_000200 *?X*) which is a subclass of XLogP descriptor (CHEMINF_000186) and was calculated using OpenEye (has_agent CHEMINF_000267). The query returns the name of the compound (*?compname*), the type of the descriptor (*?desctype*), the name of the descriptor, which is unique for each descriptor in the knowledge base (*?descname*), and the numeric descriptor value (*?descvalue*).

### Conclusions

We have introduced the Chemical Information Ontology, a formal ontology pertaining to chemical information entities that is being developed collaboratively within the context of the OBO Foundry. Our ontology allows the annotation of provenance and disambiguation of type to chemical property data being brought in ever increasing quantities onto the biological semantic web in support of whole-systems integrative research [22]. We intend the ontology to be adopted as a community standard for the widespread annotation of cheminformatics data on the semantic web, and we therefore emphasise community feedback through the provision of a mailing list and tracker and through participation in the OBO Foundry, and we welcome new use cases and requirements for ontology extension.

The domain of chemical information is a rich domain for information content entities such as descriptors and algorithms, with each software vendor providing potentially subtly different definitions and implementations of the objects of the domain. Our ontology provides multiple axes of classification through the use of OWL necessary and sufficient conditions and a DL-reasoner, allowing each of the possible axes of classification to be captured in a single ontology while avoiding a tangled asserted hierarchy. We explicitly interrelate algorithms, software implementations, descriptors and parameter values, as well as relating each descriptor to that chemical attribute that it best describes. In so doing we provide a unified and interoperable domain ontology beneath a common upper level, where previous efforts in chemical information ontology had focused on one of descriptors, algorithms, or implementations without providing a formalisation of how these entities interrelate.

Future work will involve extending the ontology to achieve closer to full coverage of known descriptors, algorithms and software vendors. With greater coverage, the CHEMINF ontology may become an asset in chemical software interoperability towards having a standard representation for chemical data. In this respect, we anticipate that the CHEMINF ontology will have a major role to play in the semantic annotation and provenance of chemical data in both the ChEBI [17] and Bio2RDF [72] projects. We further anticipate that this ontology will play a pivotal role in the establishment of chemical semantic web services towards automated chemical knowledge discovery.

## Methods

### Collaborative ontology development

Key to the development of community convergence ontologies, such as those promoted by the OBO Foundry effort, is the use of tools which allow and manage the contributions of multiple ontology editors into a single ontology. For this purpose, we have used Protégé ontology editor, version 4.0.2 [73] together with version control provided by the Google Code project [28]. Such version control provides a record of the edits which are made to the ontology file and prevents the accidental overwriting of an edited file with an earlier version being edited by a different person.

We investigated the use of Collaborative Protégé [74] for this purpose, however, Collaborative Protégé does not yet support OWL 2, and therefore we were unable to use it, although migration to this environment would be a goal for future work when the technical infrastructure renders it feasible.

### Generation of a chemical knowledge base

We programmatically transformed a set of 90 antidepressant drug molecules taken from PubChem [15] into a knowledge base together with descriptors calculated using CDK [56], Open Babel [75] and OEChem [76] software.

Molecules in PubChem are converted into instance data in the knowledge base. Each molecule is of type ‘chemical entity’, and is annotated with a label giving the PubChem identifier for that molecule. The calculated descriptors and their values are linked as attributes to their respective chemical entities. Each toolkit generates its own descriptor types and these are formally mapped to CHEMINF descriptors using the RDFS:subClassOf relation. For example, the CDK LogP descriptor is a sub-type of CHEMINF's LogP descriptor (CHEMINF_000251).

Provenance was captured in terms of the software (and version) plus parameters used to generate the descriptors.

The full knowledge base is available for download from: http://www.ebi.ac.uk/hastings/downloads/cheminfpopulated.zip.
